# Role of Peptidergic Nerve Terminals in the Skin: Reversal of Thermal Sensation by Calcitonin Gene-Related Peptide in TRPV1-Depleted Neuropathy

**DOI:** 10.1371/journal.pone.0050805

**Published:** 2012-11-27

**Authors:** Yu-Lin Hsieh, Chih-Lung Lin, Hao Chiang, Yaw-Syan Fu, June-Horng Lue, Sung-Tsang Hsieh

**Affiliations:** 1 Department of Anatomy, School of Medicine, College of Medicine, Kaohsiung Medical University, Kaohsiung, Taiwan; 2 Department of Anatomy and Cell Biology, College of Medicine, National Taiwan University, Taipei, Taiwan; 3 Department of Neurosurgery, Kaohsiung Medical University Hospital, Kaohsiung, Taiwan; 4 Faculty of Medicine, Graduate Institute of Medicine, College of Medicine, Kaohsiung Medical University, Kaohsiung, Taiwan; 5 Department of Biomedical Science and Environmental Biology, College of Science, Kaohsiung Medical University, Kaohsiung, Taiwan; 6 Department of Neurology, National Taiwan University Hospital, Taipei, Taiwan; 7 Graduate Institute of Brain and Mind Sciences, College of Medicine, National Taiwan University, Taipei, Taiwan; Kaohsiung Chang Gung Memorial Hospital, Taiwan

## Abstract

To investigate the contribution of peptidergic intraepidermal nerve fibers (IENFs) to nociceptive responses after depletion of the thermal-sensitive receptor, transient receptor potential vanilloid subtype 1 (TRPV1), we took advantage of a resiniferatoxin (RTX)-induced neuropathy which specifically affected small-diameter dorsal root ganglion (DRG) neurons and their corresponding nerve terminals in the skin. Thermal hypoalgesia (*p*<0.001) developed from RTX-treatment day 7 (RTXd7) and became normalized from RTXd56 to RTXd84. Substance P (SP)(+) and TRPV1(+) neurons were completely depleted (*p* = 0.0001 and *p*<0.0001, respectively), but RTX had a relatively minor effect on calcitonin gene-related peptide (CGRP)(+) neurons (*p* = 0.029). Accordingly, SP(+) (*p*<0.0001) and TRPV1(+) (*p* = 0.0008) IENFs were permanently depleted, but CGRP(+) IENFs (*p* = 0.012) were only transiently reduced and had recovered by RTXd84 (*p* = 0.83). The different effects of RTX on peptidergic neurons were attributed to the higher co-localization ratio of TRPV1/SP than of TRPV1/CGRP (*p* = 0.029). Thermal hypoalgesia (*p* = 0.0018) reappeared with an intraplantar injection of botulinum toxin type A (botox), and the temporal course of withdrawal latencies in the hot-plate test paralleled the innervation of CGRP(+) IENFs (*p* = 0.0003) and CGRP contents in skin (*p* = 0.01). In summary, this study demonstrated the preferential effects of RTX on depletion of SP(+) IENFs which caused thermal hypoalgesia. In contrast, the skin was reinnervated by CGRP(+) IENFs, which resulted in a normalization of nociceptive functions.

## Introduction

Neuropeptides such as calcitonin gene-related peptide (CGRP) and substance P (SP) are presumably responsible for transmitting nociceptive stimuli from sensory nerve terminals of the skin. CGRP is implicated in several painful conditions, for example elevation of CGRP in migraines [1], and increased CGRP(+) nerve fibers in tumor-bearing bone tissues [2]. The differential expression of the CGRP gene was related to variable phenotypes of thermal responses to nociceptive stimuli [3]. The reduced expression of CGRP in the spinal dorsal horn was accompanied by reduced thermal sensations [4], [5]. However, there are limited reports providing direct evidence of the role of CGRP in cutaneous nerve terminals for thermonociception after nerve degeneration.

In some patients with small-fiber sensory neuropathy, thermal hypoalgesia is associated with skin denervation [6], [7]. Nerve terminals of CGRP and SP phenotypes in the skin belong to small-diameter neurons, the soma of which are located in dorsal root ganglia (DRGs). These neurons also express transient receptor potential vanilloid subtype 1 (TRPV1) [8], which can be depleted by an ultrapotent capsaicin agonist, resiniferatoxin (RTX) [9], [10]. To investigate pathophysiology of small-fiber sensory neuropathy, we and other groups previously established a system of RTX-induced neuropathy with thermal hypoalgesia due to cutaneous nerve terminal degeneration, particularly peptidergic nerve terminals [11]–[14]. In this model, there is a lack of systematic studies investigating the relationship between TRPV1 and neuropeptides and the influence of such changes on nociceptive functions after RTX-induced neuropathy, including the peptidergic profiles in DRG neurons and molecules compensating for the loss of TRPV1(+) neurons.

Botulinum toxin type A (botox) has emerged as an effective treatment for various pain disorders including migraine control by intramuscular administration [15] and bladder cystitis therapy by intravesical treatment [16]. A potential analgesic mechanism is related to interference with neurotransmission [17]. We hypothesized that CGRP might act as a candidate molecule and asked (1) whether botox caused cutaneous nerve degeneration or only changed the expression of neuropeptides and (2) how these changes paralleled thermal responses in RTX-induced neuropathy.

**Figure 1:**
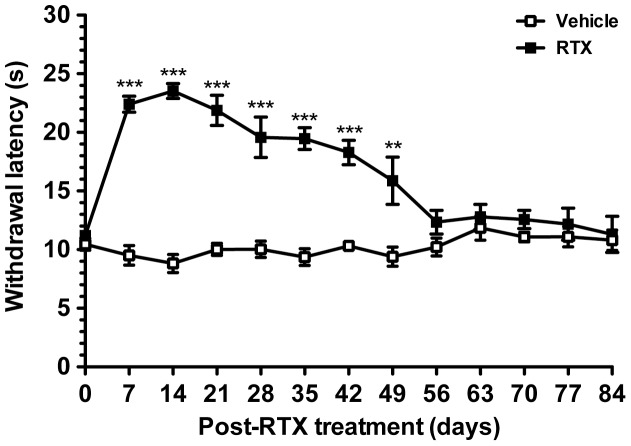
Changes in the withdrawal latency with a hot-plate test in resiniferatoxin (RTX)-induced neuropathy. Thermal responses were evaluated by the hot-plate test at 52°C in the vehicle (open squares) and RTX groups (filled squares). Withdrawal latencies remained the same in the vehicle group. Thermal hypoalgesia was induced on RTX-treatment day 7 (RTXd7, *p*<0.001) and persisted to RTXd49 (*p*<0.01), and the withdrawal latency had become normalized by RTXd56 (*p*>0.05), persisted to RTXd84 (*p*>0.05). Comparisons of hot-plate latencies (n = 5 at each time point) were analyses by two-way repeated measures ANOVA followed Bonferroni’s *post-hoc* test. *p<0.05, **p<0.01, ***p<0.001.

To address the above issues, we investigated (1) the peptidergic expression profiles of DRG neurons in relation to TRPV1, (2) the corresponding peptidergic innervation patterns of the skin, (3) nociceptive functions in relation to peptidergic innervation of the skin, and (4) the influence of botox on skin innervation in terms of peptidergic profiles of cutaneous nerve terminals.

**Figure 2:**
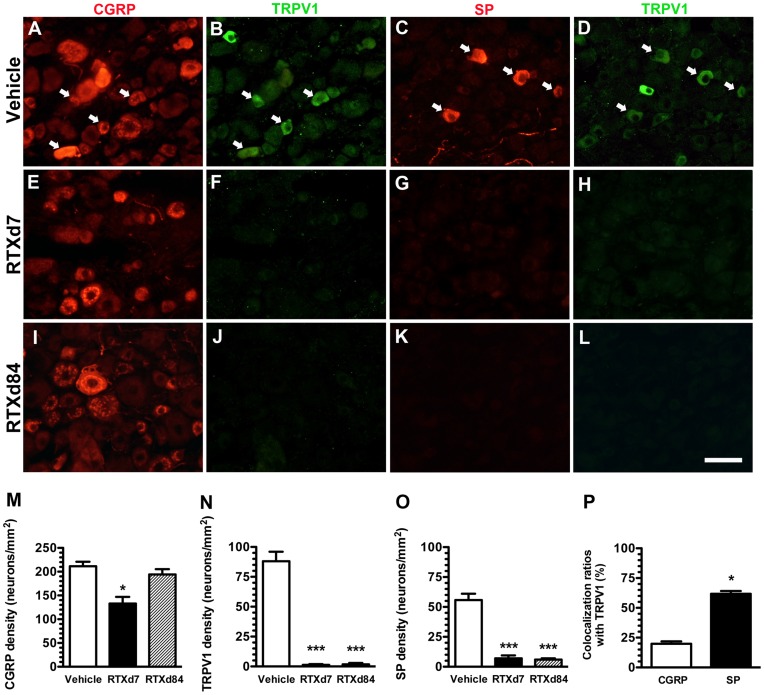
Expression patterns of peptidergic neurons in resiniferatoxin (RTX)-induced neuropathy. Double-labeling immunofluorescent staining was performed with either anti-calcitonin gene-related peptide (CGRP, A, E, I, in red) or anti-substance P (SP) (C, G, K, in red) and anti-transient receptor potential vanilloid subtype 1 (TRPV1; B, F, J, D, H, L, in green) antisera in the vehicle group (A–D) and on RTX-treatment day 7 (RTXd7) (E–H) and RTXd84 (I–L). (A–D) Some CGRP(+) neurons (A) were co-localized with TRPV1(+) neurons (B) (arrow in A and B). SP(+) and TRPV1(+) neurons (C vs. D) were highly co-localized (arrow in C and D) in the vehicle group. (E–H) CGRP(+) neurons were mild reduced (E), whereas SP(+) (G) and TRPV1(+) neurons (F, H) were completely depleted on RTXd7. (I–L) The abundance of CGRP(+) neurons was nearly normalized (I), but depletion of SP(+) (K) and TRPV1(+) neurons (J, L) remained on RTXd84. (M–O) Densities of CGRP(+) (M), TRPV1(+) (N) and SP(+) (O) neurons were quantified accordingly: vehicle group (open bars, n = 5), RTXd7 (filled bars, n = 5), and RTXd84 (gray bars, n = 5). (P) The figure compares co-localization ratios of TRPV1/CGRP (open bars, n = 5) and TRPV1/SP neurons (filled bars, n = 5). Bar, 50 µm; *p<0.05, **p<0.01, ***p<0.001.

## Materials and Methods

### Systemic RTX Treatment

RTX was used to create neuropathy in 8-week-old adult male ICR mice (35∼40 g) [14]. Briefly, RTX (Sigma, St. Louis, MO) was dissolved in a vehicle (10% Tween 80 and 10% ethanol in normal saline). Animals received a single dose of RTX by an intraperitoneal injection (50 µg/kg, the RTX group). One group received an equal volume of vehicle as the control (the vehicle group). After an intraperitoneal injection, mice were housed in plastic cages on a 12-h light/12-h dark cycle and were given access to water and food *ad libitum*. All procedures were conducted in accordance with ethical guidelines for laboratory animals [18], and the protocol was approved by the Animal Committee of National Taiwan University College of Medicine (Permit Number: 20100021) and Kaohsiung Medical University (Permit Number: 100055). All experimental procedures were performed under 4% chloral hydrate (dose: 100 g in 1 ml) except for the behavioral evaluations which were performed on awake mice. All efforts were made to minimize suffering.

**Figure 3:**
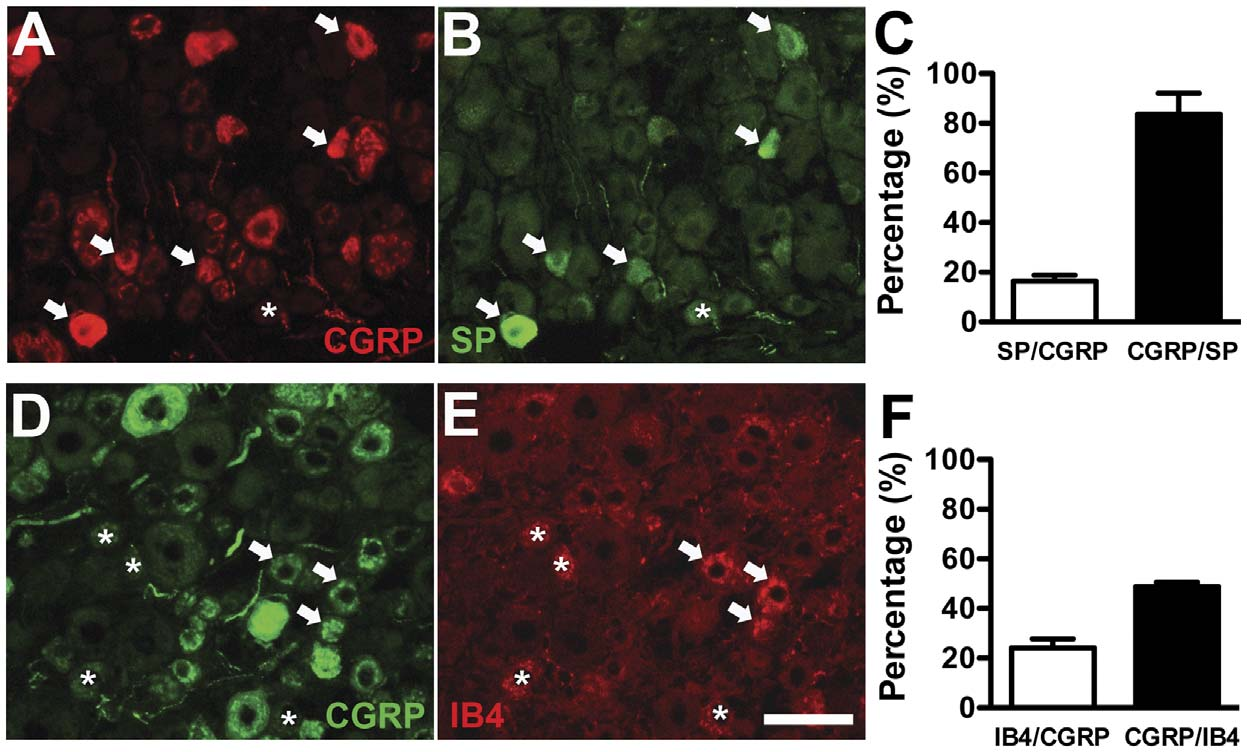
Colocalization of substance P (SP) and isolectin B4-binding (IB4) neurons with calcitonin gene-related peptide (CGRP) neurons in dorsal root ganglion (DRG). (A, B) Double-labeling immunofluorescent staining was performed with anti-CGRP (A in red) and anti-SP (B, in green) antisera on DRG sections. Some of CGRP(+) neurons were colocalized with SP(+) neurons (arrow in A and B), whereas there were CGRP(−)/SP(+) neurons (asterisk in A and B). (C) The graph shows the colocalization ratios of SP(+)/CGRP(+) (open bar, n = 5) and CGRP(+)/SP(+) neurons (filled bar, n = 5). (D, E) These graphs show the coexpression of CGRP(+) (D in green) and IB4(+) (E in red) neurons on DRG sections. There was limited colocalization of IB4(+)/CGRP(+) neurons (arrow in D, E), and there was CGRP(−)/IB4(+) neurons (asterisk in D, E). (F) The graph shows the colocalization ratio of IB4(+)/CGRP(+) (open bar, n = 5) and CGRP(+)/IB4(+) neurons (filled bar, n = 5). Bar, 50 µm.

### Evaluation by a Hot-plate Test

Thermal withdrawal latencies were measured with a hot-plate test. Briefly, mice were placed on a 52°C hot plate (IITC, Woodland Hills, CA), enclosed by a Plexiglas cage. The withdrawal latencies of the hindpaw to noxious thermal stimulations were determined to an accuracy of 0.1 s. Each test session consisted of three trials, at 30-min intervals. The criteria of withdrawal included shaking, licking, or jumping on the hot plate. The cutoff limit was 25 s to avoid potential tissue damage. The mean latency was expressed as the threshold of individual mice to the noxious thermal stimulation. Mice were subjected to the hot-plate test before RTX treatment (RTXd0), on RTX-treatment day 7 (RTXd7) and then weekly until the experimental endpoint of RTXd84.

**Figure 4:**
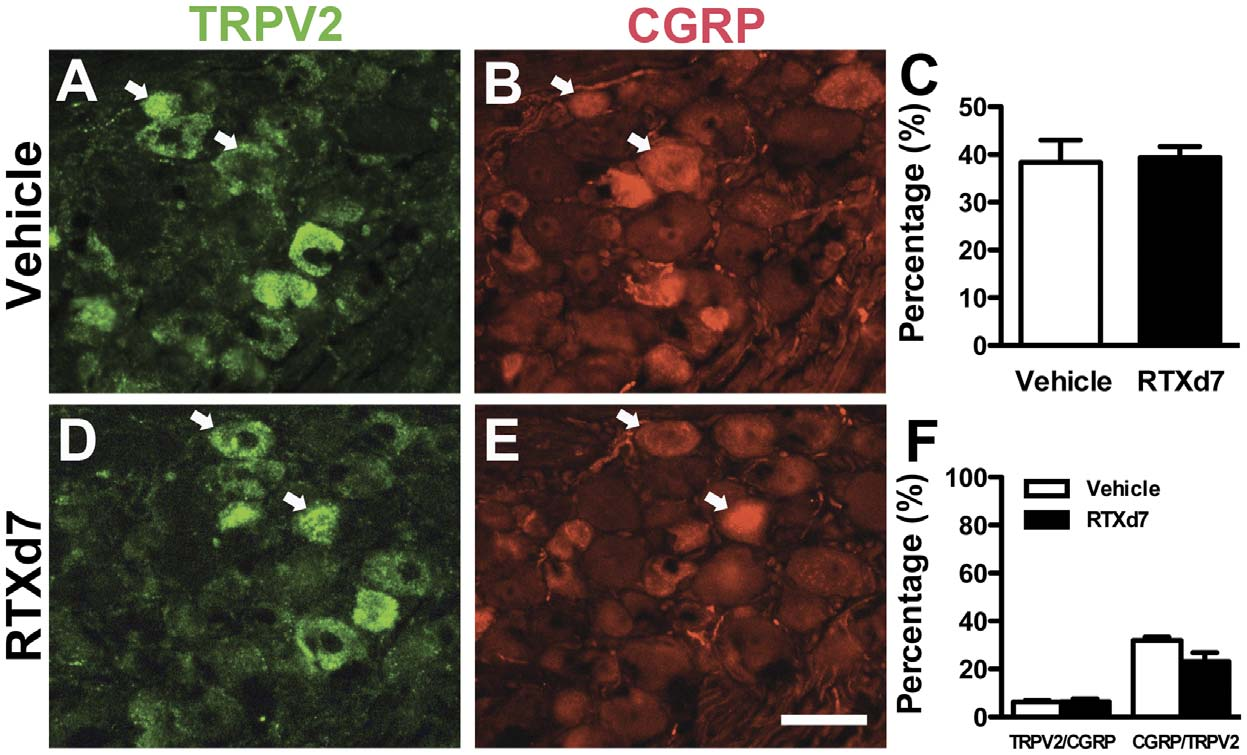
Colocalization of transient receptor potential vanilloid subtype 2 (TRPV2) and calcitonin gene-related peptide (CGRP) neurons in dorsal root ganglion (DRG). Double-labeling immunofluorescent staining was performed with anti-TRPV2 (A, D in green) and anti-CGRP (B, E in red) antisera with DRG sections of vehicle group (A, B) and on resiniferatoxin (RTX)-treatment day 7 (RTXd7) (D, E). TRPV2(+) neurons were medium-sized neurons (A, D in green) and rarely colocalized with CGRP(+) neurons (arrow in A, B, D, E). (C) The graph shows the quantification of TRPV2(+) neuron densities in the vehicle group (open bar, n = 5) and on RTXd7 (filled bar, n = 5). (F) The graph shows the colocalization ratios of TRPV2(+)/CGRP(+) and CGRP(+)/TRPV2(+) neurons in the vehicle group (open bar, n = 5) and on RTXd7 (filled bar, n = 5). Bar, 50 µm.

### Immunohistochemistry of Footpad Skin

Mice were sacrificed by intracardiac perfusion with 0.1 M phosphate buffer (PB) followed by 4% paraformaldehyde (4P) in 0.1 M phosphate buffer (pH 7.4) [19]. Footpad skin was removed for post-fixation in 4P overnight and cryosectioning with a sliding microtome at 30 µm thick. To ensure adequate sampling, every third section and a total of 10 sections for each animal were immunostained with different antisera. Primary antisera included anti-protein gene product 9.5 (PGP9.5, rabbit, 1∶2000, UltraClone, Isle of Wight, UK), SP (rabbit, 1∶2000, ImmunoStar, Hudson, WI), CGRP (rabbit, 1∶2000, Sigma), TRPV1 (goat, 1∶1000, Santa Cruz Biotechnology, Santa Cruz, CA), and receptor activity-modifying protein-1 (RAMP-1, goat, 1∶1000, Santa Cruz). Footpad skin was incubated with primary antisera overnight at 4°C, and after rinsing in 0.5 M Tris buffer (Tris), sections were incubated with corresponded biotinylated-labeled secondary immunoglobulin G (Vector, Burlingame, CA) for 1 h and the avidin-biotin complex (Vector) for another 1 h. The reaction product was demonstrated by 3,3′-diaminobenzidine (Sigma).

**Figure 5:**
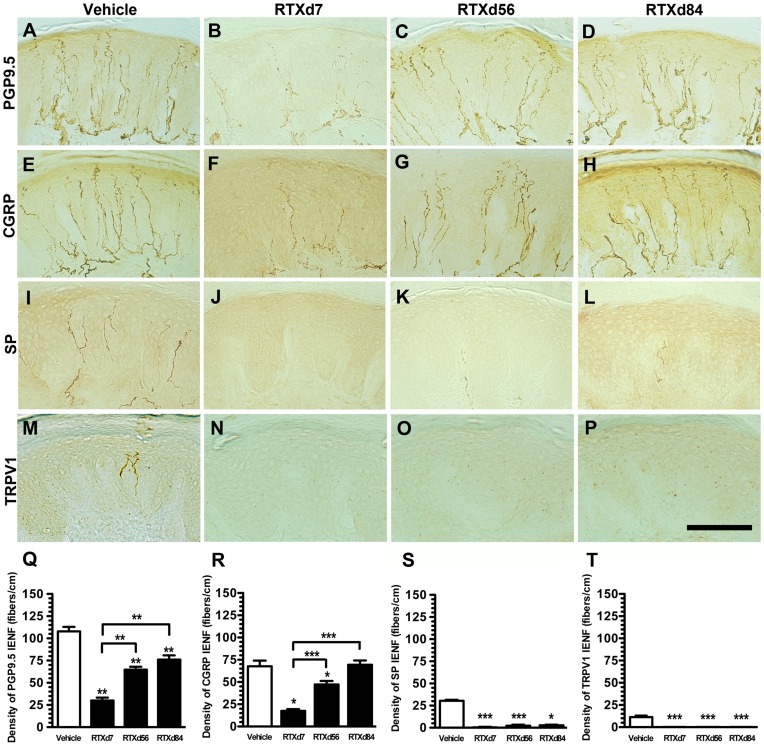
Skin innervation of different phenotypic intraepidermal nerve fibers (IENFs) in resiniferatoxin (RTX)-induced neuropathy. Different phenotypes of IENFs were demonstrated with protein gene product 9.5 (PGP9.5, A∼D), calcitonin gene-related peptide (CGRP, E∼H), substance P (SP, I∼L), and transient receptor potential vanilloid subtype 1 (TRPV1, M∼P) in the vehicle group (A, E, I, M), and on RTX-treatment day 7 (RTXd7, B, F, J, N), RTXd56 (C, G, K, O), and RTXd84 (D, H, L, P). IENFs of different phenotypes were quantified: PGP9.5(+) (Q), CGRP(+) (R), SP(+) (S) and TRPV1(+) (T) (n = 5 for vehicle group and RTXd7, n = 7 for RTXd56 and RTXd84). Bar, 100 µm; *p<0.05, **p<0.01, ***p<0.001.

For double-labeling studies, the tyramide signal amplification (TSA) technique was applied [20], [21]. Briefly, sections were incubated with the first antiserum overnight at 4°C, a biotinylated-labeled secondary antibody for 1 h, and streptavidin-horseradish peroxidase (HRP) (1∶200, PerkinElmer, Waltham, MA) for 30 min. Signals were amplified with the fluorescein tyramide reagent (1∶100, PerkinElmer) for 3 min. After rinsing in Tris, sections were incubated with the second antiserum, followed by a Texas red-conjugated secondary antibody for 1 h (1∶100, Jackson ImmunoResearch, West Grove, PA). Footpad sections were mounted on gelatin-coated slides for further analyses. Digital images of double-labeling sections were obtained at 400x magnification under Leica confocal microscope (Wetzlar, Germany).

**Figure 6:**
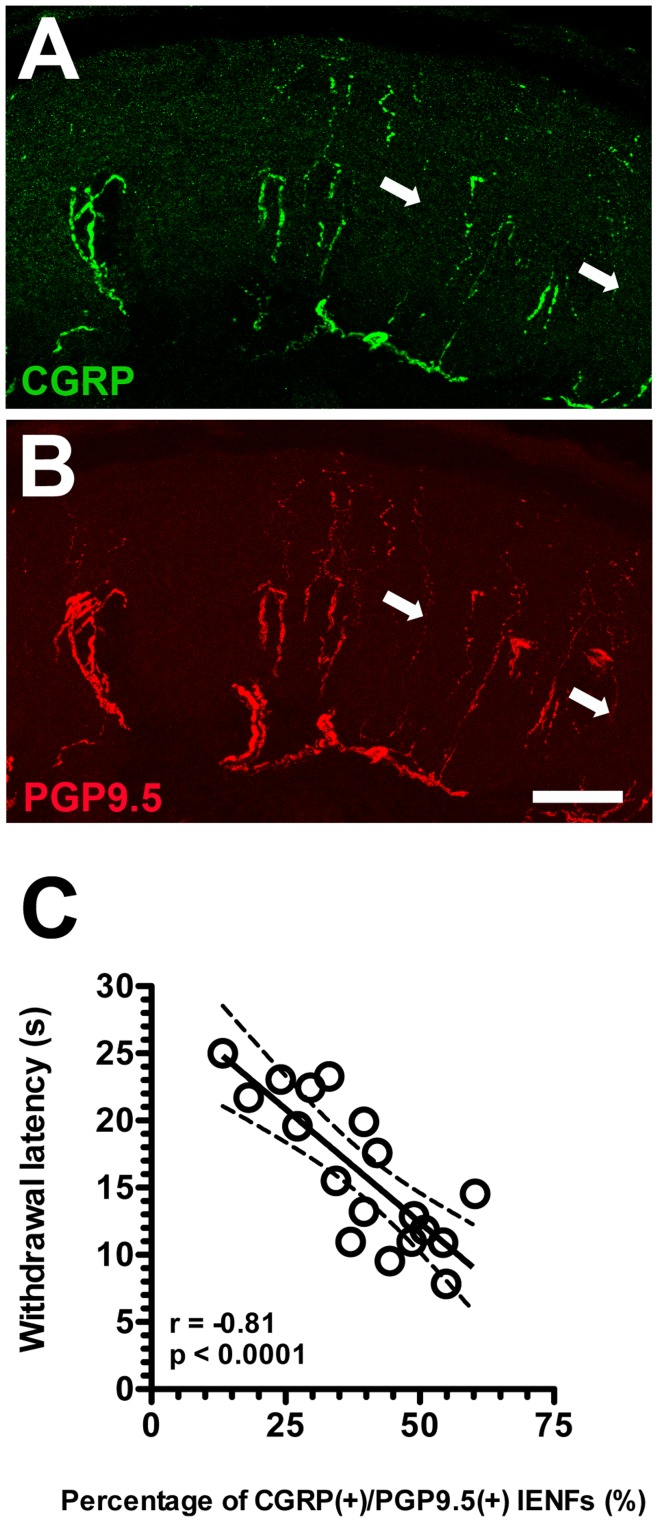
Colocalization of calcitonin gene-related peptide (CGRP) and protein gene product 9.5 (PGP9.5) in intraepidermal nerve fibers (IENFs) and the relationship with hot-plate latencies in resiniferatoxin (RTX)-induced neuropathy. (A, B) The graphs shows the double-labeling of CGRP(+) (A, in green) and PGP9.5(+) IENFs (B, in red) in the vehicle group. CGRP(+) IENFs were colocalized with PGP9.5(+) IENFs and some of PGP9.5(+) IENFs were CGRP(−) IENFs (arrow in A). (C) The graph shows the correlation of CGRP(+)/PGP9.5(+) IENFs with withdrawal latency on hot-plate test in RTX-induced neuropathy. Bar, 50 µm.

### Double-labeling Immunofluorescent Staining of DRG Neurons

For double-labeling of DRG neurons, DRG tissues were cryoprotected with 30% sucrose in PB overnight and cryosectioned with a cryostat (CM1850, Leica, Wetzlar, Germany) at 8 µm thickness. For adequate sampling, two ganglia (L4/L5) per mice and five∼eight sections per DRG tissue (at 80-µm intervals) were immunostained. Briefly, sections were incubated with one of a mixture of primary antiserum: (1) TRPV1 (1∶100)/CGRP (1∶1000) (2) TRPV1 (1∶100)/SP (1∶800) (3) CGRP (1∶1000)/SP (guinea pig, 1∶400, Peninsula Laboratories, San Carlos, CA) (4) IB4 (1∶100, Sigma)/CGRP (1∶1000) (5) TRPV2 (rabbit, 1∶1000, EMD Millipore, Billerica, MA)/CGRP (guinea pig, 1∶1400, Peninsula Laboratories) overnight at 4°C, followed by incubation with either Texas red or fluorescein isothiocyanate (FITC)-conjugated secondary antisera (1∶100, Jackson ImmunoResearch), corresponding to the appropriate primary antisera for 1 h. Sections were mounted with Vectashield (Vector) for quantification.

**Figure 7:**
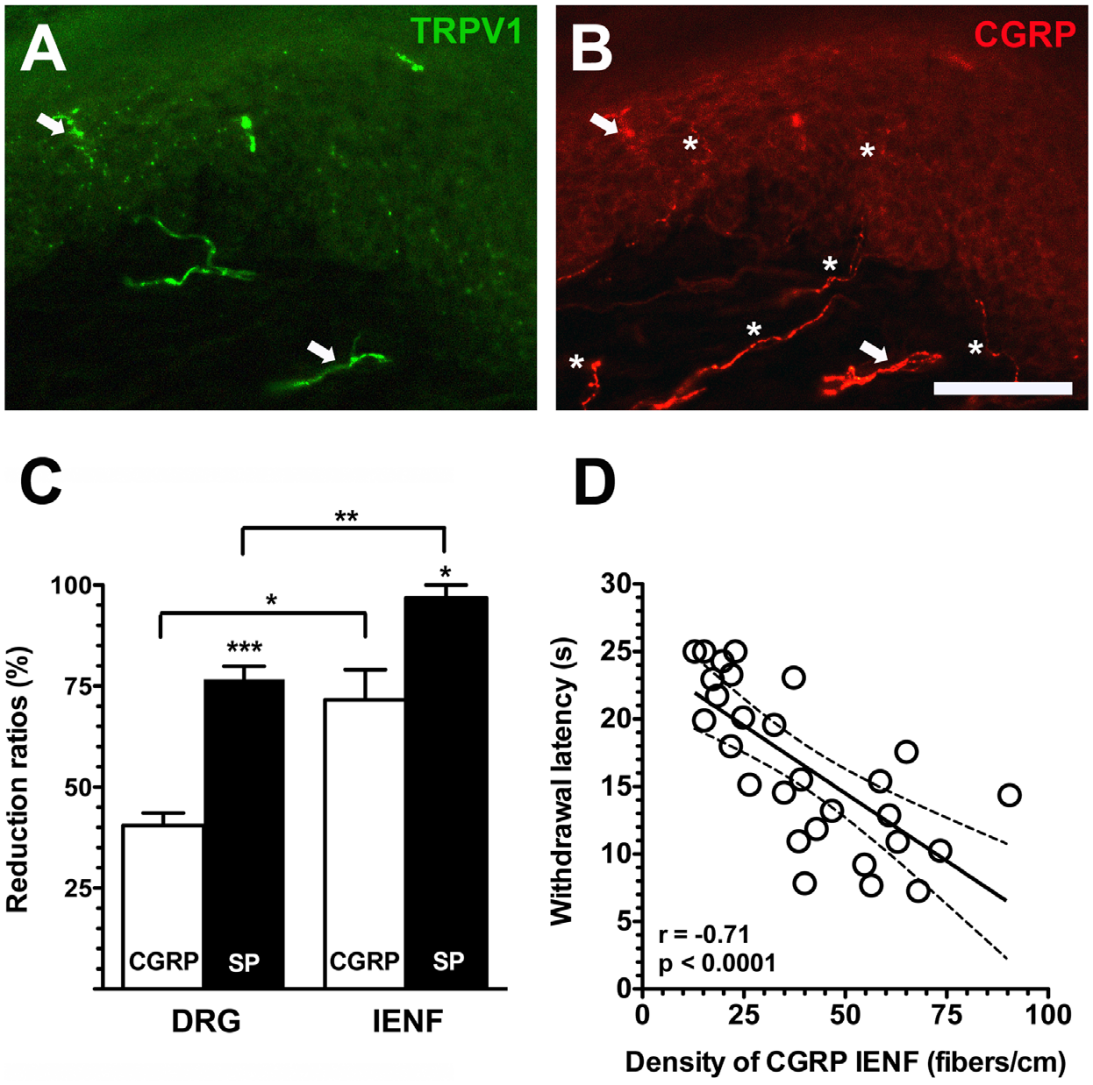
Differential extents of depletion of calcitonin gene-related peptide (CGRP) and substance P (SP) neurons and intraepidermal nerve fibers (IENFs) in resiniferatoxin (RTX)-induced neuropathy. (A, B) Double-labeling immunofluorescent staining was performed with anti-transient receptor potential vanilloid subtype 1 (TRPV1, A in green) and calcitonin gene-related peptide (CGRP, B in red) on the footpad skin of the vehicle group. There were limited CGRP(+) IENFs were coexpressed TRPV1 (arrow in A and B) and mostly CGRP(+) IENFs were TRPV1(−) IENFs (asterisk in B). (C) The graph shows different reduction ratios on the density of IENFs and dorsal root ganglia (DRGs) of different phenotypes: SP (filled bars, n = 5) and CGRP (open bars, n = 5). (D) The graph shows the correlation of CGRP(+) IENFs and withdrawal latency on hot-plate test in RTX-induced neuropathy. Bar, 100 µm; *p<0.05, **p<0.01, ***p<0.001.

### Quantification of different Phenotypic DRG Neurons and IENFs

To quantify DRG neurons of different phenotypes, each DRG section was photographed at 200x under a fluorescence microscope (Axiophot microscope, Zeiss, Oberkochen, Germany) in a systematic fashion to produce a montage of the entire DRG section following established procedures [14], [21]. To avoid a density bias, each section that only contained neuronal ganglia was measured with Image J vers. 1.44d software (National Institutes of Health (NIH), Bethesda, MD), and only neurons with a clear nuclear profile were counted. The colocalization ratios of different phenotypes DRG neurons were calculated respectively.

**Figure 8:**
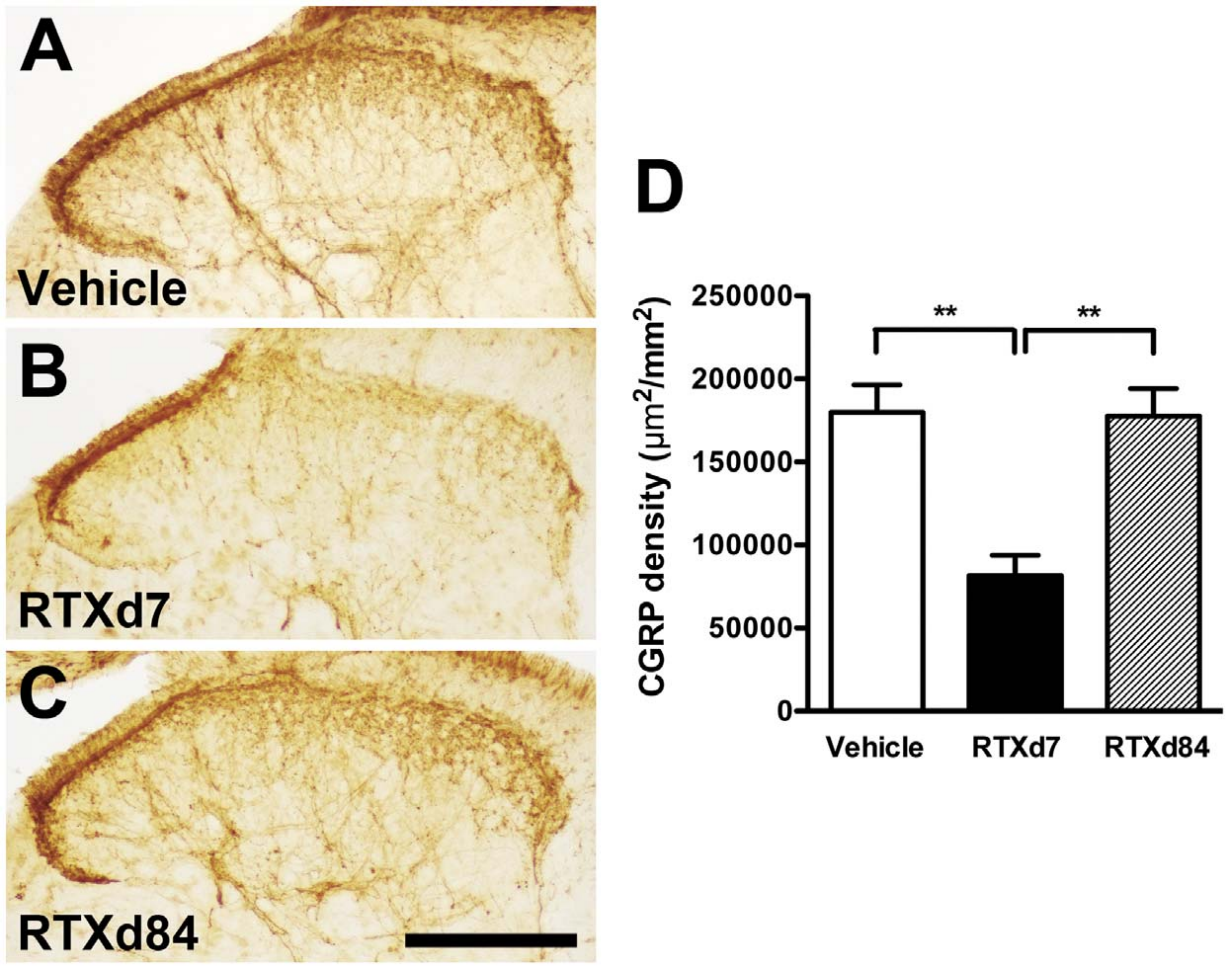
Patterns of calcitonin gene-related peptide (CGRP)(+) nerve terminals in the spinal cord of resiniferatoxin (RTX)-induced neuropathy. CGRP(+) nerve terminals were demonstrated by anti-CGRP antiserum on the lumbar cord sections and quantified. (A∼C) The graphs show the patterns of CGRP(+) nerve terminals in the vehicle group (A), and the RTX groups on day 7 (RTXd7, B) and RTXd84 (C). (D) The graph shows the quantification results of CGRP(+) nerve terminals according to Figure 8A∼C with one-way ANOVA test. (n = 5 for each group at time point). Bar, 200 µm; **p<0.01.

To quantify IENFs of different phenotypes, including PGP9.5(+), CGRP(+), SP(+), and TRPV1(+) IENFs were counted at 400x magnification (Axiophot microscope, Zeiss). The counting protocol followed established criteria in a coded fashion [22]. Fibers with branching points within the epidermis were counted as a single IENF. For fibers with branching points in the dermis, each fiber was counted as a single IENF. The length along the lower margin of the stratum corneum was defined as the epidermal length and determined with Image J software (NIH). IENF density was defined as the counted IENFs divided by the epidermal length (fibers/cm). In preliminary studies, PGP9.5(+) IENFs were 108.9±3.5 fibers/cm in the normal mice (n = 23). The values followed a Gaussian distribution (*p* = 0.27, Shapiro-Wilk normality test) and the variations among animals were minimal (3.21% of the mean).

**Figure 9:**
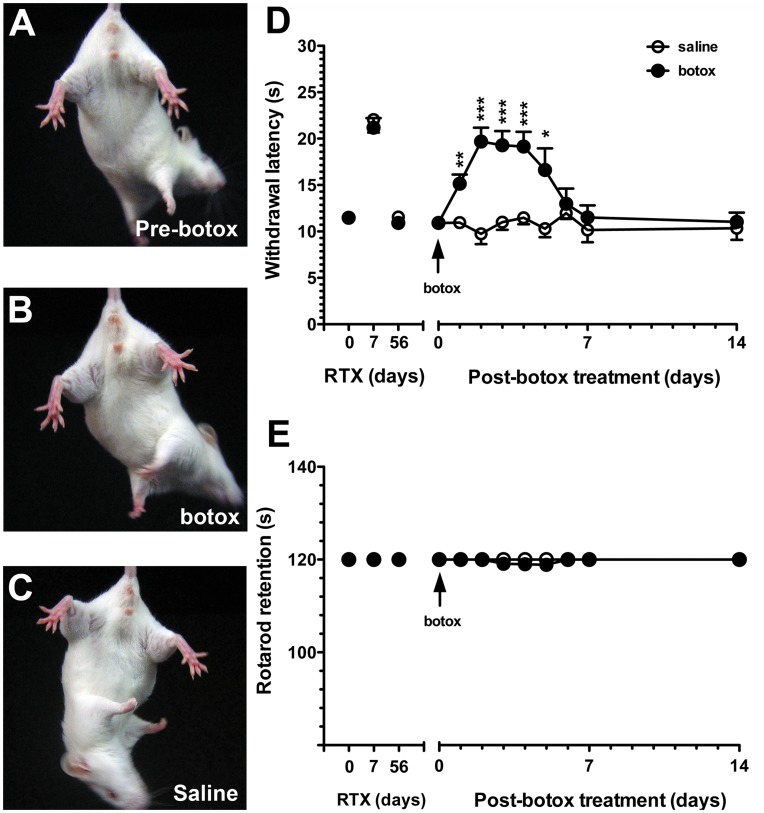
Effect of botulinum toxin type A (botox) on withdrawal latencies on the hot-plate test and motor performance in resiniferatoxin (RTX)-induced neuropathy. Botox was administered through intraplantar injection on RTX treatment day 56 (RTXd56). (A∼C) Graphs show the extension ability of hindlimbs before botox treatment (A, Pre-botox), in the group of after botox treatment (B, botox) and in the saline-treated group (C, saline). There was no difference in gross appearance of hindlimb extension among the 3 groups. (D) The graph shows the effect of botox on the withdrawal latency on the hot-plate test at 52°C in the saline group (open circles, n = 5) and botox group (filled circles, n = 5). On RTXd56, the withdrawal latency was normalized (arrow). Thermal hypoalgesia reappeared on post-botox day 1 (Bd1, *p*<0.01) and persisted until Bd5 (*p*<0.05). Hot-plate latencies between botox and saline groups were analyzed with two-way repeated measures ANOVA followed by Bonferroni’s *post-hoc* test. (E) There was no difference in retention times between the saline group (open circles, n = 5) and botox group (filled circles, n = 5) according to rotarod performance at a speed of 8 rpm (*p*>0.05). *p<0.05, **p<0.01, ***p<0.001.

The reduction ratios of CGRP(+) and SP(+) neurons and IENFs were calculated using the following formula: depletion ratio (%) = [1 - (densities of peptidergic neurons and IENFs at RTXd7/densities of peptidergic neurons and IENFs in the vehicle group)] x 100%.

**Figure 10:**
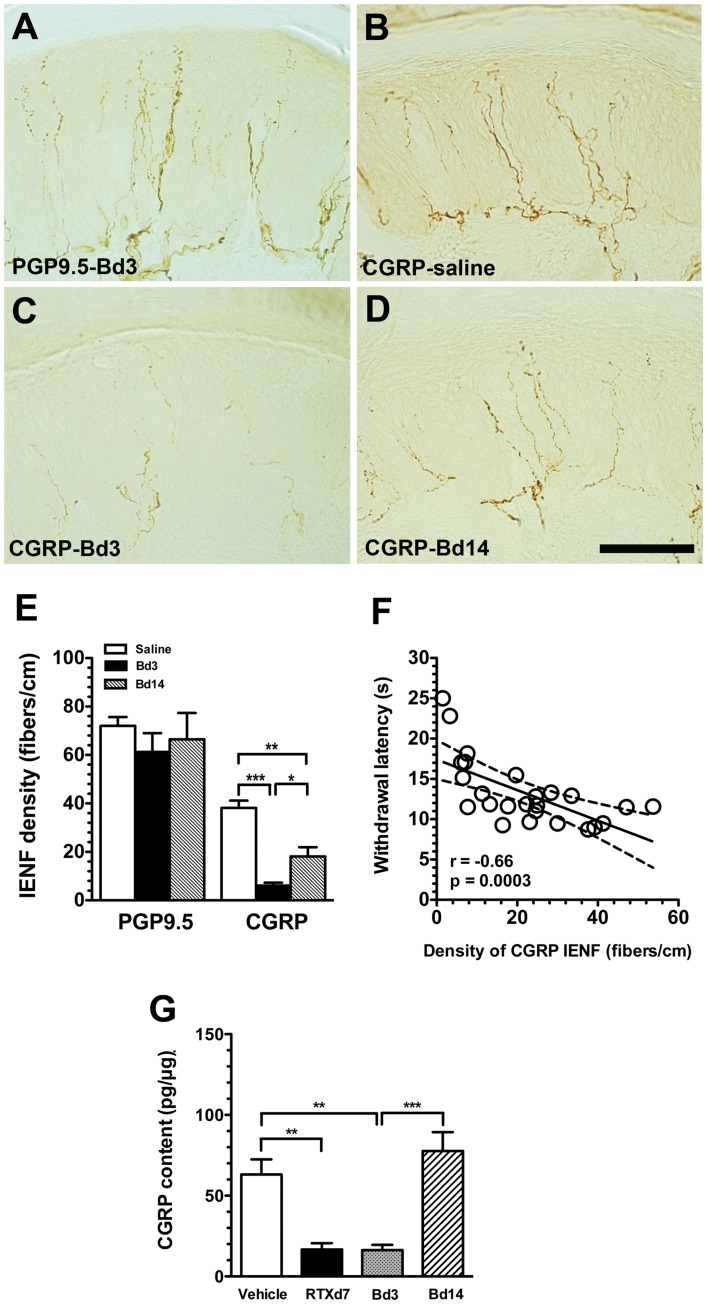
Changes in different phenotypic intraepidermal nerve fibers (IENFs) after an intraplantar injection of botulinum toxin type A (botox) on day 56 of resiniferatoxin (RTX)-induced neuropathy. (A∼D) The figure shows IENFs of protein gene product 9.5 (PGP9.5) on post-botox day 3 (Bd3, A) and calcitonin gene-related peptide (CGRP, B∼D) in the saline group (B), Bd3 (C) and Bd14 (D). (E) IENFs of PGP9.5(+) and CGRP(+) from A∼D were quantified and expressed as the density of IENFs (fibers/cm) (n = 5 for PGP9.5(+) and CGRP(+) IENFs, respectively). (F) Densities of CGRP(+) IENF were inversely correlated with withdrawal latencies on the hot-plate test. (G) The graph shows the changes in CGRP contents of skin with CGRP enzyme immunoassay (EIA) in the vehicle group (open bar, n = 5), on RTX-treatment day 7 (RTXd7) (filled bar, n = 5), Bd3 (gray bar, n = 7) and Bd14 (slash bar, n = 7). The procedures of CGRP EIA were following the manufacturer instructions and CGRP content was normalized to the protein content of epidermis. The CGRP contents were compared with the one-way ANOVA test. Bar, 100 µm; *p<0.05, **p<0.01, ***p<0.001.

### Immunohistochemistry and Quantitation of CGRP(+) Nerve Terminals in the Spinal Cord

The lumbar cord sections of 50 µm in thickness were immunostained with anti-CGRP (1∶2000) antisera as the described before. To ensure adequate sampling, every sixth section and a total of five sections for each animal were used. Sections were then mounted on slides for further quantitation [23]. Briefly, the areas of the superficial part of the dorsal horn (laminae I and II) were outlined under a dark-field microscope (Axiophot microscope, Zeiss). The areas of CGRP(+) nerves terminals on dorsal horn were quantified and normalized to the outlined areas of the superficial dorsal horn. The densities of CGRP(+) nerve terminals were expressed as µm^2^/mm^2^.

**Figure 11:**
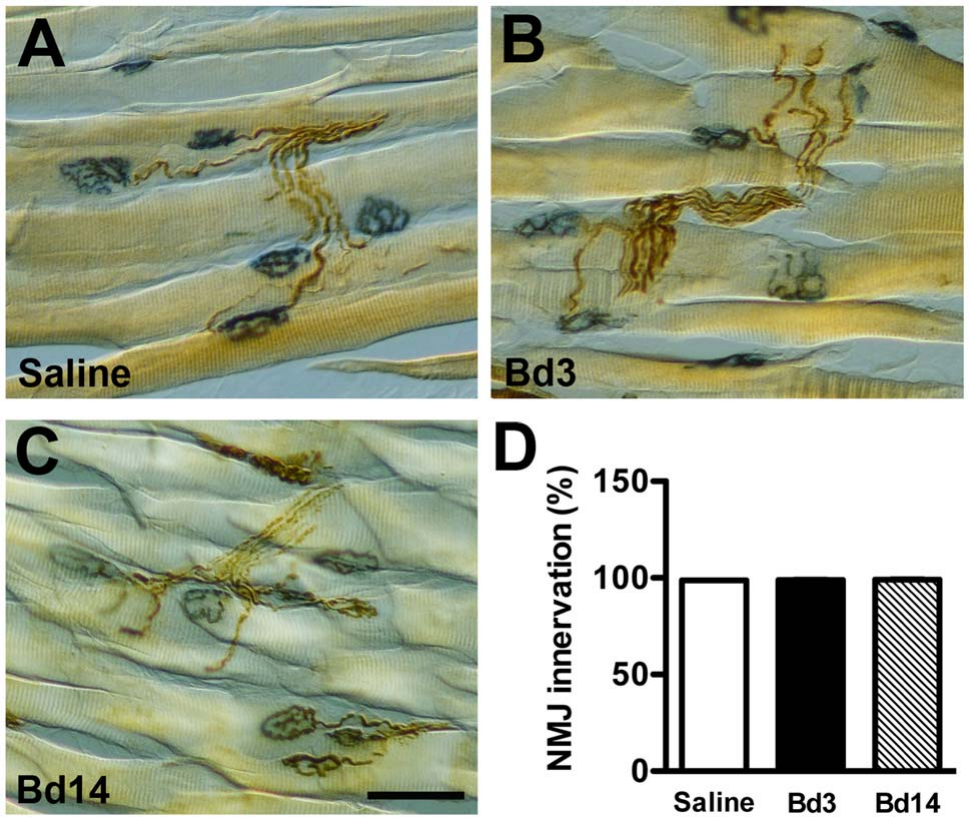
Assessment of neuromuscular junction (NMJ) innervation after an intraplantar injection of botulinum toxin type A (botox) on day 56 of resiniferatoxin (RTX)-induced neuropathy. NMJ innervation was shown by histochemistry of acetylcholine esterase and immunohistochemistry of protein gene product 9.5 (PGP9.5) in plantar muscles. (A∼C) The overlap of NMJs (blue) and PGP9.5(+) nerves (brown) indicates that the muscle was innervated in the saline group (A) and on post-botox day 3 (Bd3, B) and Bd14 (C). (D) The graph shows the quantitation of NMJ innervation ratios according to immunostaining results of 11A∼C (n = 5, *p* = 0.96, one-way ANOVA test). Bar, 50 µm.

### Intraplantar Administration of Botox after RTX-induced Neuropathy

This study investigated the role of CGRP in nociception using an intraplantar injection of botox (BOTOX®, Allergan, Irvine, CA) on RTXd56 when thermal hypoalgesia had been normalized. Briefly, botox was dissolved in sterilized saline and bilaterally administrated to the hindpaws (22.5 pg/paw in 10 µl) with a Hamilton microsyringe (Hamilton, Reno, NV). Thermal withdrawal latencies were measured by the hot-plate test as described above on post-botox day 1 (Bd1) through Bd14.

**Figure 12:**
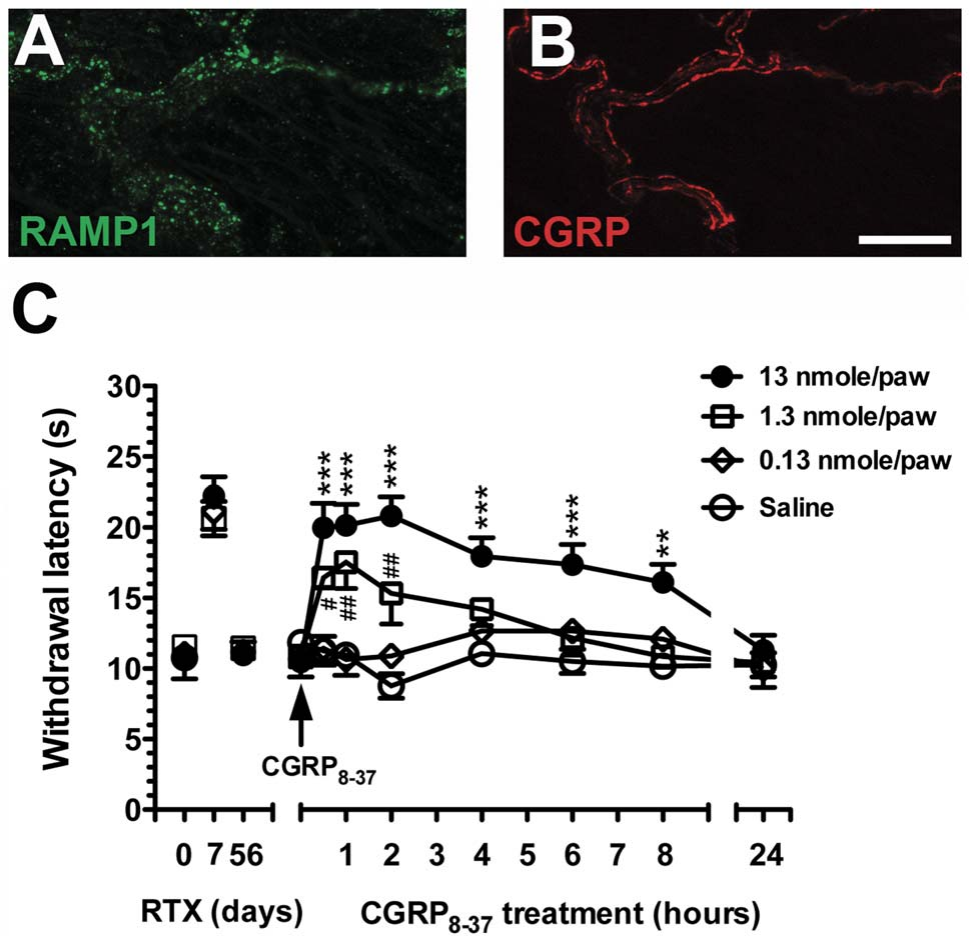
Pharmacological antagonism by an intraplantar injection of CGRP_8–37_ on day 56 of resiniferatoxin (RTX)-induced neuropathy. (A, B) The graphs show colocalization of receptor activity-modifying protein 1 (RAMP-1, A in green) and calcitonin gene-related peptide (CGRP) (B, in red) on cutaneous nerves by double-labeling immunofluorescent staining. (C) CGRP_8–37_, a CGRP antagonist, was administrated by intraplantar injections into the bilateral hindpaws. Withdrawal latencies on the hot-plate test were measured at 0.5, 1, 2, 4, 6, 8, and 24 h after antagonism. Thermal hypoalgesia was induced after antagonism in a dose-dependent manner. Hot-plate latencies of the CGRP_8–37_ antagonism group vs. the saline group were analyzed with two-way repeated measures ANOVA followed by Bonferroni’s *post-hoc* test. *p<0.05, **p<0.01, ***p<0.001, represented as CGRP_8–37_ (13 nmole/paw, n = 5) group compared to the saline group. #*p*<0.05, ##*p*<0.01, represented as CGRP_8–37_ (1.3 nmole/paw, n = 5) group compared to the saline group. Bar, 25 µm.

### Evaluation of Motor Function with a Rotarod Retention Test

To evaluate whether botox caused motor dysfunction, the rotarod retention time was measured followed a previous protocol [24]. Briefly, mice were trained to balance on a rotarod treadmill (Med Associates, Georgia, VT) at 8 rpm for 120 s. The rotarod test was performed before and after botox at each time point following the hot-plate test up to Bd14. There were three trials of the rotarod test at 20-min intervals for each mouse, and the mean of the retention times for the three trials was used for further analysis.

### Assessment of Neuromuscular Junction (NMJ) Innervation

Innervation of NMJ in the plantar muscle was evaluated with a combination of cholinesterase histochemistry and PGP9.5 immunostaining following our previously established protocols [25]. Briefly, the plantar muscles were carefully dissected and cryoprotected with 30% sucrose overnight. Series sections (30 µm) were cut on a cryostat (CM 1850, Leica). Every fifth section was stained with cholinesterase followed by PGP9.5-immunohistochemistry. Sections were incubated in a 5-bromoindoxyl acetate solution for 15 min to demonstrate the cholinesterase in NMJ followed by PGP9.5-immunohistochemistry. Coded sections from the botox and saline groups were examined, and 20 fields were randomly selected under microscopy at 200x magnification. The NMJ innervation ratios were calculated according to the number of innervated NMJ divided by the number of total NMJ on all sections.

### Pharmacological Intervention with CGRP_8–37_ by an Intraplantar Injection

Pharmacological experiments were performed with a single dose of the CGRP receptor antagonist, CGRP_8–37_ (Sigma), at various concentrations of 0.13, 1.3, and 13 nmole/paw in a volume of 10 µl on RTXd56. Drugs were dissolved in normal saline and administrated to the bilateral hindpaws with a Hamilton microsyringe (Hamilton). The withdrawal latency was measured according to the hot-plate test as described above. The other group of RTX mice received normal saline as the control for comparison. Changes in withdrawal latencies were assessed at 0.5, 1, 2, 4, 6, 8, and 24 h after CGRP_8–37_ antagonism.

### Evaluation of CGRP Contents in the Skin

To evaluate the contents of CGRP after botox, enzyme immunoassay (EIA) of CGRP was performed on the skin. Briefly, the plantar skin of the hindpaw was incubated in ethylenediaminetetraacetic acid (EDTA) solution at 37°C for 30 min. The epidermis was separated from the dermis. The epidermis was eluted in normal saline with ultrasonic bath for 30 min. The contents of CGRP were determined with the CGRP EIA kit (SPI-BIO, Massy Cedex, France) following the manufacturer’s instructions. The protein contents of epidermis were determined with the protein assay kit (Bio-Rad laboratories, Hercules, CA). The CGRP content was normalized to the protein content (pg/µg).

### Experimental Designs and Statistic Analysis

In this study, there were two experimental designs: RTX-induced neuropathy and a CGRP functional intervention, including botox and CGRP_8–37_ antagonist treatments for RTX-induced neuropathy. In the neuropathy model, there were two groups: RTX and vehicle groups. For the CGRP functional studies, there were two experiment designs: (1) the effect of botox (the botox group) and (2) the effect of CGRP antagonism (the CGRP_8–37_ group) on RTXd56. In both experimental designs, a separate group of mice received saline as a control (the saline group). Coding information was masked during the behavioral tests and quantification procedures. There were 120 mice in total with 5∼7 mice in each group at different time points for the behavioral functional evaluation and morphology examinations. All data are expressed as the mean ± standard derivation of the mean, and *t*-test was performed for data with a Gaussian distribution. For data which did not follow a Gaussian distribution, a nonparametric Mann-Whitney test was conducted. *p*<0.05 was considered statistically significant. For comparison of hot-plate latencies, two-way repeated measures ANOVAs were performed followed by Bonferroni’s *post-hoc* test when *p*<0.05 was obtained.

## Results

### Thermal Hypoalgesia with RTX-induced Neuropathy

Typical thermal hypoalgesia developed in the RTX group in comparison with the vehicle group (*p*<0.0001, two-way repeated measures ANOVA test). Before RTX treatment, the hot-plate latencies were similar between the RTX and vehicle groups (10.5±1.6 vs. 11.2±1.8 s, *p*>0.05). On RTXd7, the RTX group had marked increase in hot-plate latencies (22.4±3.5 s, *p*<0.001). Thermal hypoalgesia existed from RTXd7 to RTXd49 (15.5±5.3 s, *p*<0.01) and hot-plate withdrawal latencies had become normalized on RTXd56 (12.3±3.0 s, *p*>0.05) through RTXd84, the end point of the study period (11.3±3.5 s, *p*>0.05) (Fig. 1).

### Differential Depletion of SP(+) and CGRP(+) DRG Neurons

To understand the effects of RTX on peptidergic neurons, we examined the co-expressions of neuropeptides with TRPV1 on DRG neurons. In the vehicle group, CGRP was mainly expressed in small- to medium-sized neurons, and some CGRP(+) neurons were co-localized with TRPV1(+) neurons (Fig. 2A vs. B). Most SP(+) neurons were co-localized with TRPV1(+) neurons (Fig. 2C vs. D). On RTXd7, CGRP(+) neurons were mildly reduced (211.1±19.0 vs. 132.9±28.2 neurons/mm^2^, *p* = 0.029), and the density of CGRP(+) neurons had returned to a non-significant difference on RTXd84 (194.3±22.2 neurons/mm^2^, *p* = 0.34) (Fig. 2E, I, M). In contrast, TRPV1 was mainly expressed on small-sized neurons (88.1±16.0 neurons/mm^2^), and TRPV1(+) neurons were completely depleted on RTXd7 (1.2±1.4 neurons/mm^2^, *p*<0.0001) and RTXd84 (1.8±2.4 neurons/mm^2^, *p*<0.0001) (Fig. 2B, D, F, H, J, L, N). Similar to TRPV1(+) neurons, SP(+) neurons were depleted on RTXd7 (55.8±15.1 vs. 7.1±4.8 neurons/mm^2^, *p* = 0.0001) and RTXd84 (6.0±1.7 neurons/mm^2^, *p* = 0.0004) (Fig. 2C, G, K, O). The co-localization ratio of TRPV1(+)/SP(+) neurons was higher than that of TRPV1(+)/CGRP(+) neurons (61.9%±4.6% vs. 19.8%±4.1%, *p* = 0.029, Fig. 2P). Taken together, fewer CGRP(+) neurons were colocalized with TRPV1(+) neurons than SP(+) neurons and these were resistant to RTX-induced neuropathy.

### Colocalization of Nociceptive Molecules with CGRP(+) Neurons in RTX-induced Neuropathy

We investigated the nociceptive molecules colocalized with CGRP(+) neurons in RTX-induced neuropathy. There were limited CGRP(+) DRG neurons with co-expression of SP (16.4% ±4.8%; Fig. 3A–C) or IB4 (24.2%±6.3%; Fig. 3D–F) in the vehicle group. TRPV2 was expressed in medium-sized DRG neurons and those patterns remained the same on RTXd7 (38.4±9.2 vs. 39.4±4.0 neurons/mm^2^, *p* = 0.40) (Fig. 4 A, C, D). TRPV2(+) neurons were rarely colocalized with CGRP(+) neurons and had similar colocalization ratios in the vehicle group and the RTX group on RTXd7 (Fig. 4F).

### Influences of RTX on IENFs with different Phenotypes

To investigate the above influence of RTX on peripheral terminals of DRG neurons, we investigated IENF patterns with different markers at each time point. PGP9.5(+) IENFs emerged from the subepidermal plexus and had a typical varicose appearance within the epidermis (Fig. 5A). On RTXd7, PGP9.5(+) IENFs were markedly reduced (Fig. 5B), but these nerve fibers had gradually increased by RTXd56 and RTXd84 (Fig. 5C, D). CGRP(+) IENFs had similar innervation patterns to PGP9.5(+) IENFs at the different time points (Fig. 5E∼H). In contrast, there were only sparse SP(+) (Fig. 5I) and TRPV1(+) IENFs (Fig. 5M) in the vehicle group, and these nerve fibers were completely depleted by RTXd7, RTXd56, and RTXd84 (Fig. 5J∼L for SP and 5N∼P for TRPV1). The above observations were verified by quantitative comparisons of IENFs (Fig. 5Q∼T). Double-labeling studies indicated mostly PGP9.5(+) IENFs were coexpressed CGRP (Fig. 6A and B) and CGRP(+)/PGP9.5(+) IENFs were inversely correlated to hot-plate latencies (*r* = -0.81, *p*<0.0001) (Fig. 6C).

### Distinct Depletion Patterns of SP(+) and CGRP(+) DRG Neurons and IENFs

The different ratios of depletion in SP(+) and CGRP(+) DRG neurons were attributed to distinct patterns of colocalization with TRPV1, i.e., the difference in the co-localization ratio paralleled the higher reduction ratio of SP(+) neurons than of CGRP(+) neurons (76.7%±6.4% vs. 40.5%±6.2%, *p* = 0.0002) (Fig. 2P and 7C). Compared to the degree of depletion in peptidergic DRG neurons, the reduction ratio of SP(+) IENFs was higher than that of CGRP(+) IENFs (96.8%±6.4% vs. 71.6%±14.9%, *p* = 0.021) which resulted from low colocalization ratio of TRPV1(+)/CGRP(+) IENFs (10.2%±4.6%, Fig. 7A–C). These data also indicated that peptidergic nerve terminals were more susceptible to RTX than were neuronal soma (Fig. 7C). In summary, the neurotoxic effects of RTX were variable on IENFs of different phenotypes and resulted in different patterns of epidermal reinnervation. Further analyses indicated that densities of CGRP(+) IENFs were also correlated with hot-plate latencies (*r* = −0.71, *p*<0.0001) (Fig. 7D).

### Patterns of Dorsal Horn CGRP(+) Nerve Terminals in RTX-induced Neuropathy

Changes in the patterns of dorsal horn CGRP(+) nerve terminals paralleled those of CGRP(+) neurons and IENFs in RTX-induced neuropathy (Fig. 8). CGRP(+) nerve terminals in the dorsal horn were moderately depleted at RTXd7 compared to those of the vehicle group (179,909±33,066 vs. 81,502±24,381 µm^2^/mm^2^, *p*<0.001) (Fig. 8A vs. B). The patterns of CGRP(+) nerve terminals returned to the baseline level by RTXd84 (177,525±33,215 µm^2^/mm^2^, *p*>0.05; Fig. 8C).

### Functional Effects of Botox on Thermal Sensations

For a functional intervention of CGRP(+) IENFs, we delivered botox by an intraplantar injection on RTXd56 of RTX-induced neuropathy after thermal sensations had become normalized. Botox reduced CGRP(+) IENFs, and thermal hypoalgesia reappeared. Under the dose of botox we used (22.5 pg/paw), there was no gross change in gait or extension response of the hind limbs compared to the saline group (Fig. 9A∼C). Thermal hypoalgesia developed on Bd1 (15.1±4.2 vs. 9.7±3.1 s, *p*<0.01, two-way repeated measures ANOVA) and persisted up to Bd5 (16.6±5.2 vs. 10.3±1.8 s, *p*<0.05). Hot-plate latencies had returned to the baseline value by Bd6 (11.5±2.9 vs. 11.9±1.5 s, *p*>0.05) through Bd14 (11.0±3.0 vs. 10.4±2.2 s, *p*>0.05; Fig. 9D). Consistent with normal motor function, rotarod retention times were similar between the botox and saline groups at each time point (*p* = 1.00, Fig. 9E).

### Effects of Botox on Cutaneous Innervation of CGRP

To investigate the influence of botox on skin innervation, we assessed PGP9.5(+) and CGRP(+) IENFs. The abundance of PGP9.5(+) IENFs did not change on Bd3 (Fig. 10A). In contrast, CGRP(+) IENFs were reduced on Bd3 compared to the saline group, and there was a return of CGRP(+) IENFs on Bd14 compared to Bd3 (Fig. 10B∼D). Quantitatively, densities of CGRP(+) IENFs were markedly reduced on Bd3 (6.1±2.7 vs. 38.1±8.7 fibers/cm, *p*<0.0001), but they had recovered by Bd14 (18.1±10.8 fibers/cm, *p* = 0.023); however, the value was still lower than that of the saline group (*p* = 0.003). Densities of PGP9.5(+) IENFs were similar on Bd3 (61.3±17.3 fibers/cm, *p* = 0.19) and Bd14 (66.4±21.8 fibers/cm, *p* = 0.86), which were comparable to the saline group (72.1±12.4 fibers/cm) (Fig. 10E). In the botox experiment, densities of CGRP(+) IENFs paralleled hot-plate latencies (*r* = −0.66, *p* = 0.0003, Fig. 10F).

We then measured the changes in CGRP contents of the skin which paralleled the CGRP expression in the skin nerve terminals. On RTXd7, CGRP contents in the RTX group were markedly reduced compared to the vehicle group (16.7±7.8 vs. 63.1±18.7 pg/µg, *p*<0.01). Botox had similar effect on the reduction of CGRP content on Bd3 (16.3±7.8 pg/µg, *p* = 0.01) and CGRP contents recovered on Bd14 (77.6±23.4 pg/µg, *p*>0.05) (Fig. 10G).

### Assessment of NMJ after Botox Treatment with RTX-induced Neuropathy

NMJ innervation was examined after botox treatment. In plantar muscles of the saline group, PGP9.5 immunoreactivities appeared in nerve bundles and their branches. PGP9.5(+) motor nerve terminals were superimposed on NMJ, indicating that NMJ were innervated (Fig. 11A). On Bd3 and Bd14, patterns were similar (Fig. 11B, C). Quantitatively, NMJ innervation ratios were essentially the same among groups (*p* = 0.96; Fig. 11D).

### Pharmacological Intervention with CGRP_8–37_ Antagonism

To investigate the CGRP-mediated transmission of nociceptive stimuli, we produced a pharmacological blockade with the CGRP receptor antagonist, CGRP_8–37_, by intraplantar administration on RTXd56. RAMP-1 showed punctate profiles, and the immunoreactivities were co-expressed with CGRP immunoreactivities in cutaneous nerves of the footpad skin (Fig. 12A, B).

Before CGRP_8–37_ administration, hot-plate latencies had been normalized on RTXd56 in each group (*p* = 0.79). CGRP_8–37_ (13 nmole/paw) caused thermal hypoalgesia at 0.5 h (20.0±3.8 vs. 11.3±2.3 s, *p*<0.001, two-way repeated measures ANOVA). The effect lasted for 8 h (16.1±2.8 vs. 10.2±1.3 s, *p*<0.01), and hot-plate latencies had again become normalized by 24 h (11.3±2.4 vs. 10.3±1.9 s, *p*>0.05). The effect of CGRP_8–37_ followed a dose-dependent manner, i.e., thermal hypoalgesia only lasted for 2 h at 1.3 nmole/paw (15.3±4.4 vs. 8.8±1.9 s, *p*<0.01), and there was no effect at 0.13 nmole/paw (*p* = 0.97) (Fig. 12C).

## Discussion

The main findings of this report are that (1) RTX permanently depleted SP(+) and TRPV1(+) neurons and their cutaneous terminals, (2) peptidergic IENFs were more susceptible than their neuronal soma, and (3) CGRP(+) neurons and their cutaneous terminals were reversibly affected by RTX, and their nerve terminals were responsible for normalization of the thermal responses. The role of CGRP in cutaneous nociception was confirmed by (1) depletion of CGRP(+) cutaneous nerve terminals and reduced CGRP contents in the skin by an intraplantar injection of botox and (2) blocking of CGRP-mediated nociception by CGRP_8–37_ antagonism.

### Contribution of Co-localization with TRPV1 to different Patterns of Denervation and Reinnervation of SP(+) and CGRP(+) IENFs

The current study demonstrated that different degrees of the reinnervation of SP(+) and CGRP(+) IENFs were attributable to different co-localization ratios with TRPV1. The permanent depletion of SP(+) neurons and their cutaneous terminals resulted from a high co-localization ratio with TRPV1(+) neurons, which were depleted by RTX [9], [10]. In addition, the parallel depletion of SP(+) and TRPV1(+) neurons and their cutaneous terminals in the late stage (RTXd84) implied the possibility of permanent loss of TRPV1(+) neurons after RTX treatment. In contrast, CGRP(+) neurons and IENFs were recovered at RTXd84. What is the possible mechanism of CGRP recovery? CGRP(+) neurons had a lower level of co-expression with TRPV1(+) neurons than SP(+) neurons. Moreover, there were limited SP(+)/CGRP(+) neurons and TRPV1(+)/CGRP(+) IENFs. Those observations suggests CGRP(+) neurons account for the relative resistance of CGRP(+) neurons to RTX treatment than SP(+) neurons [26] and provide the possibility of reinnervation by CGRP(+) IENFs after RTX-induced neuropathy [27], i.e., the reinnervation in the late phase may result from the remaining CGRP(+) neurons with their CGRP(+) IENFs. The ratios of TRPV1(+)/CGRP(+) and TRPV1(+)/SP(+) neurons corresponded to the differential depletion of peptidergic DRG neurons, and such differences accounted for the basis of different extents of depletion of SP(+) and CGRP(+) IENFs.

### Distinct Functions of Peptidergic IENFs on Thermal Responses in Acute and Chronic Phases of RTX-induced Neuropathy

This study documented that CGRP(+) IENFs were sufficient for recovery of thermal sensation when TRPV1(+) and SP(+) IENFs were depleted. With RTX-induced neuropathy, thermal hypoalgesia occurred at RTXd7, corresponding to depletion of TRPV1(+) and SP(+) DRG neurons and their peripheral terminals in the skin. RTX-induced thermal hypoalgesia lasted for ∼7 weeks and became normalized at RTXd56 through RTXd84. During this period of thermal hypoalgesia, SP(+) and TRPV1(+) IENFs were depleted. The current study indicates that the reinnervation CGRP(+) IENFs in the late phase provided a compensatory mechanism for the transmission of thermonociceptive stimuli when SP(+) and TRPV1(+) IENFs were depleted. The role of thermal transmission by CGRP(+) IENFs was further confirmed by (1) botox-induced thermal hypoalgesia with a parallel decrease in CGRP(+) IENFs and CGRP contents in the skin, (2) blocking of CGRP transmission by CGRP_8–37_ antagonism.

TRPV1 and SP are known molecular mediators of thermal sensations [8], [28], [29] and are responsible for the induction of thermal hypoalgesia in RTX-induced neuropathy [11], [14]. TRP channels of various classes mediate transduction of temperature stimuli [30]–[32]. In particular, TRPV1 and TRPV2 channels are responsible for nociceptive heat [33] and have different distributions [34], [35]. In addition to TRPV1 and TRPV2, other types of TRP channels or polymodal C-nociceptors may be responsible for sensing heat when TRPV1 and TRPV2 are depleted [36], [37]. In the current study, TRPV2 was mainly expressed in medium-sized DRG neurons and rarely colocalized with CGRP. Moreover, there was no change in the densities of TRPV2(+) neurons and the ratios of TRPV2/CGRP during RTX-induced neuropathy, indicating TRPV2 are not responsible for the thermal compensation in RTX-induced neuropathy. In contrast, this study demonstrated the recovery of thermal sensation in the absence of TRPV1(+) neurons and their cutaneous terminals and provides new evidence and mechanisms for the compensatory recovery of thermal sensation by CGRP(+) IENFs. This recovery was further reversed by an intraplantar injection of botox, which depleted CGRP(+) IENFs and again resulted in thermal hypoalgesia.

In cutaneous nerve terminals, CGRP was co-localized with RAMP1, a chaperone component required for a functional CGRP receptor [38]. RAMP1 is a small single transmembrane protein but essential for binding of CGRP pharmacologically [39] and CGRP effects can be blocked by RAMP1 antagonism [40]. In this report, the colocalization of RAMP1 and CGRP on cutaneous nerves provided structural evidence of CGRP(+) IENFs for thermosensation. The effect of CGRP transmission on thermal sensations was further confirmed by CGRP antagonism with CGRP_8–37_. The antagonistic effects of CGRP_8–37_ were dose-dependent and transiently evidenced by temporal patterns of withdrawal latencies.

### Effects of Botox on CGRP Transmission in Cutaneous Nerve Terminals and Clinical Implications

This report documents the influences of botox on CGRP-mediated thermonociceptive transmission, which may provide mechanisms underlying botox-mediated pain-relieving effects. Two issues merit discussion: (1) whether botox completely damages IENFs structurally and (2) whether motor functions are impaired by an injection of botox. The reduction in CGRP(+) IENFs could be attributed to degeneration of cutaneous nerve terminals [14] or a change in the phenotypes of IENFs. Botox induced the transient reduction of CGRP(+) IENFs and PGP9.5(+) IENFs remained the same. By our observations of colocalization of CGRP(+)/PGP9.5(+) IENFs, those resulted indicating that IENFs remained structurally intact after the use of botox, but reduction CGRP after botox. Thermal hypoalgesia induced by botox was due to reduced thermonociceptive transmission, because this study documented the intactness of the motor system after the botox injection: (1) a normal gait and reflex response of the hind limbs, (2) same retention time on the rotarod test as the saline group, and (3) intact neuromuscular innervation.

Taken together, this study suggests that botox functionally changed CGRP(+) IENFs in the skin. Both downregulation and reduced release could contribute to the reduced CGRP contents in the skin. These findings provide a foundation of new therapeutic strategy for pain control by modulating GGRP releases [41], [42] and CGRP receptor at the nerve terminal of the skin. For example, transcutaneous botox administration is a easy and efficacy approach for modulating CGRP-mediated thermal nociception [43] and by developing specific antagonists of CGRP receptors without affecting cutaneous CGRP expression [40].

In summary, RTX, an ultrapotent ligand for TRPV1, permanently depleted TRPV1(+) and SP(+) neurons and their corresponding peripheral terminals in the skin, which resulted in thermal hypoalgesia. In this report, we further demonstrated that RTX had different degrees of influence on peptidergic epidermal nerves, mainly by depleting SP(+) IENFs. In contrast, CGRP(+) IENFs were more tolerant of the neurotoxicity of RTX and were responsible for normalization of the thermal responses in the late phase.
